# Supplementary material for: Examining Mammalian facial behavior using Facial Action Coding Systems (FACS) and combinatorics
Source: PLoS One. 2025 Jan 27;20(1):e0314896. doi: 10.1371/journal.pone.0314896 (PMC11771922; doi:10.1371/journal.pone.0314896)
Supplement: S1 Text — Please see below for more information about each file. (DOCX) [file pone.0314896.s001.docx]

Datasets (for chimpanzees and domesticated cats) and Python code can be found in the online electronic supplement (files S1-S4).
