# Supplementary material for: Examining Mammalian facial behavior using Facial Action Coding Systems (FACS) and combinatorics
Source: PLoS One. 2025 Jan 27;20(1):e0314896. doi: 10.1371/journal.pone.0314896 (PMC11771922; doi:10.1371/journal.pone.0314896)
Supplement: S1 File — This document contains supplementary tables and detailed information about the Excel workbook where our raw data is stored (S2 File, below). (DOCX) [file pone.0314896.s002.docx]

**Using Facial Action Coding Systems and Data Engineering to Evaluate the Communicative Potential in Mammals**

**Table S1.** A list of all facial muscle movements considered in our current study, as described in the chimpFACS^1,2^ and catFACS^2,3^.

| **Chimpanzees** | | **Domesticated Cats** | |
| --- | --- | --- | --- |
| **FACS Code** | **Description** | **FACS Code** | **Description** |
| AU 1+2 | Brow Raiser | AU5 | Upper Lid Raiser |
| AU 6 | Cheek Raiser | AU143 | Eyes Closed |
| AU 43 | Eye Closure | AU145 | Blink |
| AU 45 | Blink | AU47 | Half Blink |
| AU 9 | Nose Wrinkler | AD48 | Third eyelid show |
| AU 10 | Upper Lip Raiser | AD68 | Pupil dilator |
| AU 12 | Lip Corner Puller | AD69 | Pupil constrictor |
| AU 16 | Lower Lip Depressor | AU109+110 | Nose Wrinkler & Upper Lip Raiser |
| AU 17 | Chin Raiser | AU12 | Lip Corner Puller |
| AU 22 | Lip Funneler | AU116 | Lower Lip Depressor |
| AU 24 | Lip Presser | AU17 | Chin Raiser |
| AU 25 | Lips Parted | AU118 | Lip Puckerer |
| AU 26 | Jaw Drop | AU25 | Lips Part |
| AU 27 | Mouth Stretch | AU26 | Jaw Drop |
| AU 28 | Lips suck | AU27 | Mouth Stretch |
| AD 19 | Tongue Show | AD19 | Tongue Out |
| AD 21 | Neck Tightener | AD37 | Lip Wipe |
| AD 29 | Jaw Thrust | AD137 | Nose Lick |
| AD 30 | Jaw Sideways | AD190 | Tongue downwards |
| AD 32 | Bite | AU200 | Whisker retractor |
| AD 33 | Blow | AU201 | Whisker protractor |
| AD 35 | Cheek Suck | AU202 | Whisker raiser |
| AD 37 | Lip Wipe | EAD101 | Ears forward |
| AD 160 | Lower Lip Relax | EAD102 | Ear adductor |
|  |  | EAD103 | Ear flattener |
|  |  | EAD104 | Ear rotator |
|  |  | EAD105 | Ears downward |
|  |  | EAD106 | Ears backwards |
|  |  | EAD107 | Ears constrictor |

**Table S2.** A list of all facial muscle movement combinations identified in previous studies with chimpanzees^4^ and domesticated cats^5^. For both mammals, there are over 200 morphologically distinct facial muscle movement combinations identified. To access the table with all the documented combinations, please refer to the "*Mahmoud et al. Data*" workbook's excel sheet named "*Documented Combos*".

**Table S3.** A list of revised facial muscle movement combinations used in our current study. This updated list consists solely of Action Units (AUs) and Action Descriptors (ADs). For both mammals, there are over 200 morphologically distinct facial muscle movement combinations identified. To access the table with all the documented combinations, please refer to the "*Mahmoud et al. Data*" workbook's excel sheet named "*Revised Combos*".

**Table S4.** A list of rules for facial muscle movement combinations in chimpanzees. We based our research on previously documented combination rules and written descriptions of facial muscle movements found in the chimpFACS^1,2^. In cases where combination rules were unclear or absent, we referred to the humanFACS^6,7^ manual for additional guidance (for example, confirming that it is possible for AU28 and AD32 could appear together in certain situations).

| **FACS Code** | **Rules** |
| --- | --- |
| AU 1+2 | AU1 must be coded with AU2.  AU2 must be coded with AU1. |
| AU 6 | -- |
| AU 43 | AU43 cannot appear with AU45. |
| AU 45 | AU45 cannot appear with AU43. |
| AU 9 | -- |
| AU 10 | -- |
| AU 12 | -- |
| AU 16 | AU16 cannot appear with AU17.  AU16 must be coded with AU25.  AU16 cannot appear with AU22.  AU16 cannot appear with AD160. |
| AU 17 | AU17 cannot appear with AU16.  AU17 cannot appear with AD19.  AU17 cannot appear with AU22.  AU17 cannot appear with AU25.  AU17 cannot appear with AU26.  AU17 cannot appear with AU27. |
| AU 22 | AU22 cannot appear with AU16.  AU22 cannot appear with AU17.  AU22 must be coded with AU25.  AU22 cannot appear with AD160. |
| AU 24 | AU24 cannot appear with AD19.  AU24 cannot appear with AU25.  AU24 cannot appear with AU26.  AU24 cannot appear with AU27.  AU24 cannot appear with AD32. |
| AU 25 | AU25 cannot appear with AU24.  AU25 cannot appear with AU17. |
| AU 26 | AU26 cannot appear with AU27.  AU26 cannot appear with AU17.  AU26 cannot appear with AU24. |
| AU 27 | AU27 must be coded with AU25.  AU27 cannot appear with AU26.  AU27 cannot appear with AU17.  AU27 cannot appear with AU24. |
| AU 28 | AU28 must be coded with AU26.  AU28 cannot appear with AD35. |
| AD 19 | AD19 must be coded with AU25.  AD19 must be coded with AU26 or AU27.  AD19 cannot appear with AU17.  AD19 cannot appear with AU24. |
| AD 21 | -- |
| AD 29 | -- |
| AD 30 | -- |
| AD 32 | AD32 cannot appear with AU24.  AD32 cannot appear with AD35. |
| AD 33 | -- |
| AD 35 | AD35 cannot appear with AU28.  AD35 cannot appear with AU32. |
| AD 37 | AD37 must be coded with AU25.  AD37 must be coded with AU26 or AU27.  AD37 cannot appear with AU24. |
| AD 160 | AD160 cannot appear with AU16.  AD160 cannot appear with AU17.  AD160 cannot appear with AU22.  AD160 must be coded with AU25.  AD160 cannot appear with AU24. |

**Table S5.** A list of rules for facial muscle movement combinations in domesticated cats. We based our research on previously documented combination rules and written descriptions of facial muscle movements found in the catFACS^2,3^. In cases where combination rules were unclear or absent, we referred to the humanFACS^6,7^ manual for additional guidance.

| **FACS Code** | **Rules** |
| --- | --- |
| AU5 | AU5 cannot appear with AU47.  AU5 cannot appear with AU143.  AU5 cannot appear with AU145. |
| AU143 | AU143 cannot appear with AU5.  AU143 cannot appear with AU145.  AU143 cannot appear with AU47. |
| AU145 | AU145 cannot appear with AU5.  AU145 cannot appear with AU143.  AU145 cannot appear with AU47. |
| AU47 | AU47 cannot appear with AU5.  AU47 cannot appear with AU143.  AU47 cannot appear with AU145. |
| AD48 | -- |
| AD68 | AD68 cannot appear with AD69. |
| AD69 | AD69 cannot appear with AD68. |
| AU109+110 | AU109 must be coded with AU110.  AU110 must be coded with AU109. |
| AU12 | AU12 cannot appear with AU118. |
| AU116 | AU116 cannot appear with AU17.  AU116 must be coded with AU25. |
| AU17 | AU17 cannot appear with AU116.  AU17 cannot appear with AU25.  AU17 cannot appear with AU26.  AU17 cannot appear with AU27.  AU17 cannot appear with AD19.  AU17 cannot appear with AD190.  AU17 cannot appear with AD37.  AU17 cannot appear with AD137. |
| AU118 | AU118 cannot appear with AU12. |
| AU25 | AU25 cannot appear with AU17. |
| AU26 | AU26 cannot appear with AU27.  AU26 cannot appear with AU17. |
| AU27 | AU27 must be coded with AU25.  AU27 cannot appear with AU26.  AU27 cannot appear with AU17. |
| AD19 | AD19 must be coded with AU25.  AD19 must be coded with AU26 or AU27.  AD19 cannot appear with AU17.  AD19 cannot appear with AD190. |
| AD37 | AD37 must be coded with AU25.  AD37 must be coded with AU26 or AU27.  AD37 cannot appear with AU17.  AD37 cannot appear with AD137. |
| AD137 | AD137 cannot appear with AD37.  AD137 must be coded with AU25.  AD137 must be coded with AU26 or AU27.  AD137 cannot appear with AU17. |
| AD190 | AD190 must be coded with AU25.  AD190 must be coded with AU26 or AU27.  AD190 cannot appear with AU17.  AD190 cannot appear with AD19. |
| AU200 | AU200 cannot appear with AU201. |
| AU201 | AU201 cannot appear with AU200. |
| AU202 | -- |
| EAD101 | EAD101 cannot appear with EAD106.  EAD101 cannot appear with EAD103. |
| EAD102 | EAD102 cannot appear with EAD105.  EAD102 cannot appear with EAD107. |
| EAD103 | EAD103 cannot appear with EAD101.  EAD103 cannot appear with EAD106. |
| EAD104 | EAD104 cannot appear with EAD106.  EAD104 cannot appear with EAD107. |
| EAD105 | EAD105 cannot appear with EAD102.  EAD105 cannot appear with EAD106.  EAD105 cannot appear with EAD107. |
| EAD106 | EAD106 cannot appear with EAD101.  EAD106 cannot appear with EAD103.  EAD106 cannot appear with EAD104.  EAD106 cannot appear with EAD105. |
| EAD107 | EAD107 cannot appear with EAD102.  EAD107 cannot appear with EAD104.  EAD107 cannot appear with EAD105. |

**Raw data can be found in S2_File. Information about each excel sheet in this workbook can be found below:**

**S2_File (Sheet 1 – Documented Combos).** Raw data on the facial configurations of chimpanzees and domesticated from our two previous studies^4,5^.

**S2_File (Sheet 2 – Revised Combos).** Cleaned data that only includes Action Units (AUs) and Action Descriptors (ADs) for chimpanzees and domesticated cats.

**S2_File (Sheet 3 – Observed for Chimps).** A detailed list of all morphologically distinct facial signals that were identified by our data-engineered model for chimpanzees and have also been documented in previous studies^4^.

**S2_File (Sheet 4 – Observed for Cats).** A detailed list of all morphologically distinct facial signals that were identified by our data-engineered model for domesticated cats and have also been documented in previous studies^5^.

**S2_File (Sheet 5 – Flagged for Chimps).** A list of all morphologically distinct facial signals that were identified in previous chimpanzee studies^4^ but were flagged as problematic by our data-engineered model.

**S2_File (Sheet 6 – Flagged for Cats).** A list of all morphologically distinct facial signals that were identified in previous domesticated cat studies^5^ but were flagged as problematic by our data-engineered model.

**S2_File (Sheet 7 – Unobserved for Chimps).** A detailed list of all morphologically distinct facial signals that were identified by our data-engineered model for chimpanzees but have not been documented in previous studies.

**S2_File (Sheet 8 – Unobserved for Cats).** A detailed list of all morphologically distinct facial signals that were identified by our data-engineered model for domesticated cats but have not been documented in previous studies.

**References**

1 Vick, S.-J., Waller, B. M., Parr, L. A., Smith Pasqualini, M. C. & Bard, K. A. A Cross-species Comparison of Facial Morphology and Movement in Humans and Chimpanzees Using the Facial Action Coding System (FACS). *J Nonverbal Behav* **31**, 1-20 (2007). <https://doi.org/10.1007/s10919-006-0017-z>

2 Waller, B. M., Julle-Daniere, E. & Micheletta, J. Measuring the evolution of facial ‘expression’ using multi-species FACS. *Neuroscience & Biobehavioral Reviews* **113**, 1-11 (2020). <https://doi.org/https://doi.org/10.1016/j.neubiorev.2020.02.031>

3 Caeiro, C., Waller, B. & Burrows, A.

4 Florkiewicz, B. N. O., L. S.; Oña, L.; Campbell, M. W. Primate Socio-Ecology Shapes the Evolution of Distinctive Facial Repertoires. *Journal of Comparative Psychology* (2023). <https://doi.org/https://doi.org/10.1037/com0000350>

5 Scott, L. & Florkiewicz, B. N. Feline faces: Unraveling the social function of domestic cat facial signals. *Behavioural Processes* **213**, 104959 (2023). <https://doi.org/https://doi.org/10.1016/j.beproc.2023.104959>

6 Ekman, P. & Friesen, W. V.

7 Ekman, P. & Rosenberg, E. L. *What the Face Reveals: Basic and Applied Studies of Spontaneous Expression Using the Facial Action Coding System (FACS)*. (New York: Oxford University Press, 2005).
