# Supplementary material for: Examining Mammalian facial behavior using Facial Action Coding Systems (FACS) and combinatorics
Source: PLoS One. 2025 Jan 27;20(1):e0314896. doi: 10.1371/journal.pone.0314896 (PMC11771922; doi:10.1371/journal.pone.0314896)
Supplement: S3 File — This PDF contains information about the Python code used to generate our list of possible combinations for chimpanzees. (PDF) [file pone.0314896.s004.pdf]

Using Facial Action Coding Systems and Data Engineering to Evaluate the Communicative Potential in Mammals

Mahmoud et al.

This is the Google Colab notebook accompanying the manuscript as an electronic supplement. It contains the Python programming portion of the research.

CHIMPANZEE EDITION!

Background:

Mammals can create various facial expressions by combining different facial muscle movements. Each muscle movement, or action unit (AU), is assigned a number. The goal of this project is to compile an inventory of all potential facial muscle movement combinations in mammals (such as dogs, cats, and non-human primates).

For this specific program, we focused on Chimpanzees. This program algorithm will later be applied to other datasets of AUs for other mammals.

The chimpanzee data is from *Primate Socio-Ecology Shapes the Evolution of Distinctive Facial Repertoires* (Florkiewicz).

Method:

This program generates all possible combinations of AUs while respecting species-specific limitations, ensuring that biologically implausible combinations are excluded.

It uses a pre-defined set of rules and a list of the AUs to generate all feasible combinations. It then compares all these combos against a dataset of the combos that have been already observed by researchers. It outputs a list of those possible combinations that have been observed, ones that haven't been observed yet, and ones that have been observed but have been found not to be possible by the computer.

Results overview:

The total runtime of this project is about 2 minutes. It generates 238,079 posssible combinations. 238,022 have not been observed, and 57 of them have been observed. 9 of the observed combinations have been flagged as not possible according to the rules inputted, which is discussed in the manuscript.

Code begins.

Import packages.

```
import itertools
import pandas as pd
import time
```

Part 1: Defining functions!

This first function `generate_combinatorial_subsets()` creates a list of combinations and checks it against a set of predefined rules (in the `rules()` function). If the combination satisfies all the rules, then it appends it to a list named `subsets`.

```
def generate_combinatorial_subsets(items, rule):
    subsets = []
    n = len(items)
    for r in range(1, n + 1):
        for combination in itertools.combinations(items, r):
            if rules(combination):
                subsets.append(combination)
                #print(f"Appending: {combination}")  # Add this line to print before appending
    return subsets
```

The `rules` function has all the species-specific limitations encoded, to check each combination against. These rules come from the Chimpanzee FACS manual (Vick, S.-J., Waller, B. M., Parr, L. A., Smith Pasqualini, M. C., Bard, K. A.)

Chimpanzee rules:

```
total_start = time.time()
```

```
def rules(combination):

    #AU 1 must be coded with 2 and vice versa
    if 1 in combination and 2 not in combination:
        return False
    elif 2 in combination and 1 not in combination:
        return False

    #AU 43 cannot appear with 45
    if (43 in combination and 45 in combination):
        return False

    #AU 16 must be coded with 25, and cannot appear with 17, 22, or 160
    if any((16 in combination and x in combination) for x in (17, 22, 160)):
        return False
    if 16 in combination and 25 not in combination:
        return False

    #AU 17 cannot appear with 19, 22, 25, 26, 27, 160
    if any((17 in combination and x in combination) for x in (19, 22, 25, 26, 27, 160)):
        return False

    #AU 19 must be coded with 25, must be coded with 26 or 27, cannot appear with 17, 24
    if (19 in combination and 25 not in combination):
        return False
    if 19 in combination and not (26 in combination or 27 in combination):
         return False

    #AU 22 must be coded with AU 25
    if (22 in combination and 25 not in combination):
        return False

    #AU 24 cannot appear with AU 19, 25, 26, 27, 32
    if any((24 in combination and x in combination) for x in (19, 25, 26, 27, 32)):
        return False

    #AU 26 cannot appear with 17, 27, 24
    if (26 in combination and 27 in combination):
        return False

    #AU 27 must be coded with 25, and cannot appear with 26, 17, 24
    if (27 in combination and 25 not in combination):
        return False

    #AU 28 must be coded with 26, and 28 cannot appear with 35
    if (28 in combination and 26 not in combination):
        return False
    if (28 in combination and 35 in combination):
        return False

    #AU 32 cannot appear with 24 or 35
    if (32 in combination and 35 in combination):
        return False


    #AU 37 must be with 25, must be with 26 or 27
    if (37 in combination and 25 not in combination):
        return False
    if 37 in combination and not (26 in combination or 27 in combination):
         return False


    #AU 160 cannot appear with 16, 17, 22, 24, and it must be coded with 25
    if (160 in combination and 22 in combination):
        return False
    if (160 in combination and 25 not in combination):
        return False


    else: #The combination is valid
        return True
```

The `keep_only_integers()` function is used for formatting purposes. It takes the string that was read in from the dataset of observed AUs (for example, `"6+12+16+25+M69+S101"`, then splits it into each term `[6, 12, 16, 25, M69, S101]` and keeps integer-only values so the combo now reads as `[6, 12, 16, 25]`.

```
def keep_only_integers(AU_column):
  AU_column_split = [x.split('+') for x in AU_column]
  AU_column_ints = [[int(x) if x.isdigit() else x for x in y] for y in AU_column_split]
  AU_column_ints_only = [[int(x) for x in y if x.isdigit()] for y in AU_column_split]
  AU_column_ints_only_unique = list(set(tuple(x) for x in AU_column_ints_only))
  AU_column_ints_only_unique.sort()
  AU_column_ints_only_unique.sort(key=len)
  final_AU_observed = [x for x in AU_column_ints_only_unique if x] #gets rid of empty elements
  return final_AU_observed
```

```
[illegible]
```

The `string_format()` function is another function that is used for formatting purposes. It takes a list of integers `[10+12+16+25+26]` and reformats it as a connected string `"10+12+16+25+26"`.

```
def string_format(list_of_integers):
  concatenated_data = []
  concatenated_data = ['+'.join(map(str, row)) for row in list_of_integers]
  return concatenated_data
```

Part 2: Reading in observed AUs.

We then read in a .csv file that contains AU combinations that have been observed on chimpanzees (Florkiewicz, B. N. O., L. S.; Oña, L.; Campbell, M. W.), and store it in a data frame named `observed`.

This program assumes that:

1. All AU combinations are in the first column of the .csv file.
2. Each AU combination is in ascending numerical order of AUs.

```
url = "https://raw.githubusercontent.com/aishmeister/FACSCombinatorics/main/observed_datasets/observed_chimps.csv"
given_table = pd.read_csv(url)
print(given_table)
```

```
   Chimpanzee AU Combinations
0                          17
1                       17+24
2                       22+25
3                       25+26
4                       25+27
..                        ...
64                   17+22+25
65                  22+25+160
66                   24+25+26
67                16+22+25+26
68               22+25+26+160

[69 rows x 1 columns]
```

```
#keep only first column, drop all NAs from the Excel sheet
AU_unfiltered_column = given_table[given_table.columns[0]].dropna()

#split row into each term, delete terms that have letters, therefore keeping only integer combos
AU_observed_integers = keep_only_integers(AU_unfiltered_column)

#format these integer back into a string (10,4,6) --> ('10+4+6')
AU_observed = string_format(AU_observed_integers)

observed = pd.DataFrame(AU_observed)
observed.columns = ['given_AUs']
```

```
observed
```

	given_AUs
**0**	17
**1**	22
**2**	17+24
**3**	17+25
**4**	22+25
**...**	...
**61**	10+12+16+25+26+43
**62**	10+12+16+25+27+43
**63**	6+9+10+12+16+25+26
**64**	6+10+12+16+25+26+43
**65**	9+10+12+16+25+27+43

66 rows × 1 columns

PART 3: Generating combinations

Each item is an action unit (AU) that corresponds to a facial muscle movement. The `generate_combinatorial_subsets()` function calculates all the possible AU combinations that can theoretically be produced based on the set of rules given, and stores it in a list named `subsets`.

GENERATING

```
AUs = [1,2,6,43,45,9,10,12,16,17,22,24,25,26,27,28,19,21,29,30,32,33,35,37,160]
word_bank = sorted(AUs)
len(word_bank)
```

```
25
```

```
#keeping track of how long it takes to run
start = time.time()
```

```
subsets = []
subsets = generate_combinatorial_subsets(word_bank, rules)
```

```
end = time.time()
print(f"Time taken: {end - start:.2f} seconds")
```

```
Time taken: 47.06 seconds
```

```
print('The function has calculated that there are: ' + str(len(subsets)) + " possible combinations")
```

```
The function has calculated that there are: 238079 possible combinations
```

The following snippet of code turns elements in the `subsets` list into a concatenated string.

`(15, 20, 22, 26, 28, 31)` in `subsets` turns to `'15+20+22+26+28+31'` in `concatenated_data`.

```
start = time.time()
```

```
concatenated_data = string_format(subsets)
```

```
end = time.time()
print(f"Time taken: {end - start:.2f} seconds")
```

```
Time taken: 0.55 seconds
```

```
start = time.time()
```

```
combos = pd.DataFrame(concatenated_data)
combos.columns = ['combinations']
```

```
end = time.time()
print(f"Time taken: {end - start:.2f} seconds")
```

```
Time taken: 0.04 seconds
```

PART 3: Comparing

Now that the list of possible generated combinations is in a data frame named `combos`, and our list of already observed AUs is in a data frame named `observed`, we can now continue to look at how many of the possible combinations that have actually been observed in on-field observations of chimpanzees.

Checking out the structure of `observed` and `combos`:

```
observed.head()
```

	given_AUs
0	17
1	22
2	17+24
3	17+25
4	22+25

```
combos.head()
```

	combinations
**0**	6
**1**	9
**2**	10
**3**	12
**4**	17

The following block of code checks matches between `combos` and `observed`. If there's a match, it removes it from the `combos` (generated subsets) list. This way, as it removes all the seen combinations, we are left with a remaining list of all the combinations that have NOT been observed in the wild yet.

After checking equivalence, we break from the `if` check because there is no need to check a certain combo with all the rest of the combos when its match has already been found.

```
start = time.time()
```

```
unseen = []
seen = []

# Creates a copy of combos['combinations'] to work with so we don't modify the original list
remaining_combos = combos['combinations'].copy()
remaining_combos = remaining_combos.tolist()

# Comparing and dividing into list of seen and unseen AUs
for x in combos['combinations']:
    match_found = False
    for y in observed["given_AUs"]:
        if x == y:
            #print("\nPossible combo: " + str(x) + " is present in the list of observed AUS (aka we've seen it in the wild). A match with: ", str(y))
            seen.append(x)
            remaining_combos.remove(x)
            match_found = True
            break
```

```
end = time.time()
print(f"Time taken: {end - start:.2f} seconds")
```

```
Time taken: 4.40 seconds
```

We will now check for observations that have been flagged as not having a match in the generated combinations subsets, so that we may investigate them. They will be stored in a list named `observed_but_not_possible`.

```
observed_combinations = observed["given_AUs"].tolist()
generated_combinations = combos['combinations'].tolist()

observed_but_not_possible = []

for obs in observed_combinations:
    if obs not in generated_combinations:
        observed_but_not_possible.append(obs)

print("Number of observed combinations not found in generated combos:", len(observed_but_not_possible))
```

```
Number of observed combinations not found in generated combos: 9
```

```
observed_but_not_possible
```

```
['22',
 '17+25',
 '22+33',
 '17+22+25',
 '22+25+160',
 '24+25+26',
 '16+22+25+26',
 '16+22+25+27',
 '22+25+26+160']
```

```
len(remaining_combos)
```

```
238022
```

```
unseen = remaining_combos
```

```
print("length of total generated subsets (combos): " + str(len(combos)))
print("length of observed combos: " + str(len(observed)))
print("\nunseen: " + str(len(unseen)))
print("seen: " + str(len(seen)))
print("observed combos that \"don't exist\": " + str(len(observed_but_not_possible)))
```

```
length of total generated subsets (combos): 238079
length of observed combos: 66

unseen: 238022
seen: 57
observed combos that "don't exist": 9
```

```
print("Percentage of seen combos out of all possible combos: " + str(round(len(seen) / len(combos) * 100, 2)) + "%")
print("(This is " + str(len(seen)) +" out of " + str(len(combos)) + ")")

print("\nThe first 5 combos seen are: ")
print(seen[0:5])

print("\nThe first 10 combos not seen are: ")
print(unseen[0:10])
```

```
Percentage of seen combos out of all possible combos: 0.02%
(This is 57 out of 238079)

The first 5 combos seen are:
['17', '17+24', '22+25', '25+26', '25+27']

The first 10 combos not seen are:
['6', '9', '10', '12', '21', '24', '25', '26', '29', '30']
```

Next, each list is turned into a data frame so it can be exported.

```
seen_df = pd.DataFrame(seen, columns=['seen_combos'])
seen_df.to_excel('Chimps-seen_combos.xlsx', index=False)
```

```
unseen_df = pd.DataFrame(unseen, columns=['unseen_combos'])
unseen_df.to_excel('Chimps-unseen_combos.xlsx', index=False)
```

```
observed_but_not_possible_df = pd.DataFrame(observed_but_not_possible, columns=['observed_but_not_in_subsets'])
observed_but_not_possible_df.to_excel('Chimps-flagged.xlsx', index=False)
```

```
total_end = time.time()
print(f"Time taken: {total_end - total_start:.2f} seconds ({(total_end - total_start)/60:.2f} minutes)")
```

```
Time taken: 60.32 seconds (1.01 minutes)
```

PART 4 - Databases!

Next, generated subsets (`combos`), observed AUs (`observed`), `seen`, and `unseen` are uploaded into separate SQLite3 databases so they can be queried.

```
import sqlite3
```

COMBOS database

```
db_name = 'generated_combos.db'
conn = sqlite3.connect(db_name)

table_name = 'all_possible_subsets'
combos.to_sql(table_name, conn, index=False, if_exists='replace')
```

```
238079
```

```
query = "SELECT * FROM all_possible_subsets WHERE combinations LIKE '%10%'"
possibles = pd.read_sql_query(query, conn)
print(possibles)
```

```
                                          combinations
0                                                   10
1                                                 6+10
2                                                 9+10
3                                                10+12
4                                                10+17
...                                                ...
119035  1+2+6+9+10+12+16+19+21+25+26+28+29+30+32+33+37+45
119036  1+2+6+9+10+12+19+21+22+25+26+28+29+30+32+33+37+43
119037  1+2+6+9+10+12+19+21+22+25+26+28+29+30+32+33+37+45
119038  1+2+6+9+10+12+19+21+25+26+28+29+30+32+33+37+43...
119039  1+2+6+9+10+12+19+21+25+26+28+29+30+32+33+37+45...
```

```
[119040 rows x 1 columns]
```

OBSERVED AU database

```
db_name = 'observed_data.db'
conn = sqlite3.connect(db_name)

table_name = 'observed_AUs'
observed.to_sql(table_name, conn, index=False, if_exists='replace')
```

```
66
```

```
query = "SELECT * FROM observed_AUs LIMIT 10"
observeds = pd.read_sql_query(query, conn)
print(observeds)
```

```
  given_AUs
0        17
1        22
2     17+24
3     17+25
4     22+25
5     22+33
6     25+26
7     25+27
8  10+12+25
9  12+16+25
```

SEEN database

```
db_name = 'seen_combos.db'
conn = sqlite3.connect(db_name)

table_name = 'seen'
seen_df.to_sql(table_name, conn, index=False, if_exists='replace')
```

```
57
```

```
query = "SELECT * FROM seen LIMIT 10"
seen_sql = pd.read_sql_query(query, conn)
print(seen_sql)
```

```
  seen_combos
0          17
1       17+24
2       22+25
3       25+26
4       25+27
5    10+12+25
6    12+16+25
7    12+22+25
8    12+25+26
9    12+25+27
```

UNSEEN database

```
db_name = 'unseen_combos.db'
conn = sqlite3.connect(db_name)

table_name = 'unseen'
unseen_df.to_sql(table_name, conn, index=False, if_exists='replace')
```

```
238022
```

```
query = "SELECT * FROM unseen LIMIT 5"
unseen_top_5 = pd.read_sql_query(query, conn)
print(unseen_top_5)
```

```
  unseen_combos
0             6
1             9
2            10
3            12
4            21
```

```
query = "SELECT * FROM unseen WHERE unseen_combos LIKE '%5%' AND unseen_combos LIKE '%15%'"
both_5_and_15 = pd.read_sql_query(query, conn)
print(both_5_and_15)
```

```
Empty DataFrame
Columns: [unseen_combos]
Index: []
```

Results

Refer to *Using Facial Action Coding Systems and Data Engineering to Evaluate the Communicative Potential in Mammals* (Mahmoud, et al.) to read more about the results.

```
print("Chimpanzees\n")
print("length of total generated subsets: " + str(len(combos)))
print("length of observed combos: " + str(len(observed)))
print("\nunobserved: " + str(len(unseen)))
print("observed: " + str(len(seen)))
print("flagged combos: " + str(len(observed_but_not_possible)))
```

```
Chimpanzees

length of total generated subsets: 238079
length of observed combos: 66

unobserved: 238022
observed: 57
flagged combos: 9
```

References:

Birkenkrahe, Marcus. Saving large data sets to Excel. Gist. Retrieved February 9, 2024, from https://gist.github.com/birkenkrahe/57142fc39cf7b174bee597825126c83f

Florkiewicz, B. N. O., L. S.; Oña, L.; Campbell, M. W. Primate Socio-Ecology Shapes the Evolution of Distinctive Facial Repertoires. Journal of Comparative Psychology (2023). https://doi.org:https://doi.org/10.1037/com0000350

Vick, S.-J., Waller, B. M., Parr, L. A., Smith Pasqualini, M. C., Bard, K. A. A Cross- species Comparison of Facial Morphology and Movement in Humans and Chimpanzees Using the Facial Action Coding System (FACS). J Nonverbal Behav 31, 1-20 (2007). https://doi.org:10.1007/s10919-006-0017-z
