# Supplementary material for: Examining Mammalian facial behavior using Facial Action Coding Systems (FACS) and combinatorics
Source: PLoS One. 2025 Jan 27;20(1):e0314896. doi: 10.1371/journal.pone.0314896 (PMC11771922; doi:10.1371/journal.pone.0314896)
Supplement: S4 File — This PDF contains information about the Python code used to generate our list of possible combinations for domesticated cats. (PDF) [file pone.0314896.s005.pdf]

Using Facial Action Coding Systems and Data Engineering to Evaluate the Communicative Potential in Mammals

Mahmoud et al.

This is the Google Colab notebook accompanying the manuscript as an electronic supplement. It contains the Python programming portion of the research.

CATS EDITION!

Background:

Mammals can create various facial expressions by combining different facial muscle movements. Each muscle movement, or action unit (AU), is assigned a number. The goal of this project is to compile an inventory of all potential facial muscle movement combinations in mammals (such as dogs, cats, and non-human primates).

This specific program is generated for cat AUs!

The cat data is from *Feline faces: Unraveling the social function of domestic cat facial signals* (Scott, Florkiewicz).

Method:

This program generates all possible combinations of AUs while respecting species-specific limitations, ensuring that biologically implausible combinations are excluded.

It uses a pre-defined set of rules and a list of the AUs to generate all feasible combinations. It then compares all these combos against a dataset of the combos that have been already observed by researchers. It outputs a list of those possible combinations that have been observed, ones that haven't been observed yet, and ones that have been observed but have been found not to be possible by the computer.

Cat Results

The total runtime of this project is about 21 minutes. It generates 1,062,719 possible combinations. 1,062,447 have not been observed, and 272 of them have been observed. 3 of the observed combinations have been flagged as not possible according to the rules inputted, which is discussed in the manuscript.

Code begins.

Import packages.

```
import itertools
import pandas as pd
import time
```

Part 1: Defining functions!

This first function `generate_combinatorial_subsets()` creates a list of combinations and checks it against a list of predefined rules (in the `rules()` function). If the combination satisfies all the rules, then it appends it to a list named `subsets`.

```
def generate_combinatorial_subsets(items, rule):
    subsets = []
    n = len(items)
    for r in range(1, n + 1):
        for combination in itertools.combinations(items, r):
            if rule(combination):
                subsets.append(combination)
                #print(f"Appending: {combination}")  # Add this line to print before appending
    return subsets
```

The `rules` function has all the species-specific limitations encoded, to check each combination against. These rules come from the CatFACS manual (Caeiro, C. C., Burrows, A. M. & Waller, B. M.).

Cat rules:

```
def rules(combination):

    #AU 5 cannot be coded with 47, 143, 145
```

```
    if any((5 in combination and x in combination) for x in (47, 143, 145)):
        return False

    #AU 12 cannot appear with 118
    if 12 in combination and 118 in combination:
        return False

    #AU 17 cannot appear with 116, 25, 26, 27, 19, 190, 37, 137
    if any((17 in combination and x in combination) for x in (116, 25, 26, 27, 19, 190, 37, 137)):
        return False

    #AD 19 must be coded with 25, with either 26 or 27, and it cannot appear with 17, 190
    if 19 in combination and 25 not in combination:
        return False
    if 19 in combination and not (26 in combination or 27 in combination):
         return False
    if 19 in combination and 190 in combination:
        return False


    #AU 26 cannot appear with 27
    if 26 in combination and 27 in combination:
        return False

    #AU 27 cannot appear with 26 or 17, and it must be coded with 25
    if 27 in combination and 25 not in combination:
        return False

    #AD 37 must be coded with 25, with 26 or 27, cannot appear with 17, 137
    if 37 in combination and 25 not in combination:
        return False
    if 37 in combination and not (26 in combination or 27 in combination):
         return False
    if 37 in combination and 137 in combination:
        return False

    #AU 68 cannot appear with 69
    if 68 in combination and 69 in combination:
        return False

    #EAD 101 cannot appear with 103 or 106
    if 101 in combination and 103 in combination:
        return False

    #EAD 102 cannot appear with 105, 107
    if 102 in combination and 105 in combination:
        return False


    #EAD 106 cannot appear with 101, 103, 104, 105
    if any((106 in combination and x in combination) for x in (101, 103, 104, 105)):
        return False

    #EAD 107 cannot appear with 102, 104, 105
    if any((107 in combination and x in combination) for x in (102, 104, 105)):
        return False


    #AU 109 must be coded with 110
    if 109 in combination and 110 not in combination:
        return False

    #AU 110 must be coded with 109
    if 110 in combination and 109 not in combination:
        return False

    #AU 116 cannot appear with 17, must be coded with 25
    if 116 in combination and 25 not in combination:
        return False

    #AD 137 must be coded with 25, with 26 or 27, cannot appear with 17, 37
    if 137 in combination and 25 not in combination:
        return False
    if 137 in combination and not (26 in combination or 27 in combination):
         return False



    #AU 143 cannot appear with 5, 145, 47
    if any((143 in combination and x in combination) for x in (47, 145)):
        return False

    #AU 145 cannot appear with 5, 143, 47
    if 145 in combination and 47 in combination:
        return False

    #AD 190 must be coded with 25, with 26 or 27, cannot appear with 17, 19
    if 190 in combination and 25 not in combination:
```

```
        return False
    if 190 in combination and not (26 in combination or 27 in combination):
         return False

    #AU 200 cannot appear with 201
    if 200 in combination and 201 in combination:
        return False


    else: #The combination is valid
        return True
```

The `keep_only_integers()` function is used for formatting purposes. It takes the string that was read in from the dataset of observed AUs (for example, `"6+12+16+25+M69+S101"`, then splits it into each term `[6, 12, 16, 25, M69, S101]` and keeps integer-only values so the combo now reads as `[6, 12, 16, 25].`

```
def keep_only_integers(AU_column):
  AU_column_split = [x.split('+') for x in AU_column]
  AU_column_ints = [[int(x) if x.isdigit() else x for x in y] for y in AU_column_split]
  AU_column_ints_only = [[int(x) for x in y if x.isdigit()] for y in AU_column_split]
  AU_column_ints_only_unique = list(set(tuple(x) for x in AU_column_ints_only))
  AU_column_ints_only_unique.sort()
  AU_column_ints_only_unique.sort(key=len)
  final_AU_observed = [x for x in AU_column_ints_only_unique if x] #gets rid of empty elements
  return final_AU_observed
```

The `string_format()` function is another function that is used for formatting purposes. It takes a list of integers `[101+109+110+143]` and reformats it as a connected string `"101+109+110+143"`.

```
def string_format(list_of_integers):
  concatenated_data = []
  concatenated_data = ['+'.join(map(str, row)) for row in list_of_integers]
  return concatenated_data
```

Part 2: Reading in observed AUs.

We then read in a .csv file that contains AU combinations that have been observed on cats (Scott, L., Florkiewicz, B. N.), and store it in a data frame named `observed`.

This program assumes that:

1. All AU combinations are in the first column of the .csv file.
2. Each AU combination is in ascending numerical order of AUs.

```
url = "https://raw.githubusercontent.com/aishmeister/FACSCombinatorics/main/observed_datasets/observed_cats.csv"
given_table = pd.read_csv(url)
print(given_table)
```

```
     Domesticated Cat AU Combinations
0                                  47
1                                 101
2                             101+102
3                 101+102+109+110+143
4                     101+102+118+201
..                                ...
270                        69+101+200
271                            69+102
272                        69+103+104
273                            69+104
274                        69+104+105

[275 rows x 1 columns]
```

```
#keep only first column, drop all NAs from the Excel sheet
AU_unfiltered_column = given_table[given_table.columns[0]].dropna()

#split row into each term, delete terms that have letters, therefore keeping only integer combos
AU_observed_integers = keep_only_integers(AU_unfiltered_column)

#reput these integers into a string (10,4,6) --> ('10+4+6')
AU_observed = string_format(AU_observed_integers)

observed = pd.DataFrame(AU_observed)
observed.columns = ['given_AUs']
```

```
observed
```

	given_AUs
**0**	47
**1**	101
**2**	102
**3**	103
**4**	104
**...**	...
**270**	12+25+26+68+104+105+109+110+116+202
**271**	12+25+26+101+102+109+110+116+143+201
**272**	12+25+27+68+103+104+109+110+116+201
**273**	5+12+25+26+69+103+104+109+110+116+202
**274**	12+25+26+47+69+103+104+109+110+116+202

275 rows × 1 columns

PART 3: Generating combinations

Each item is an action unit (AU) that corresponds to a facial muscle movement. The `generate_combinatorial_subsets()` function calculates all the possible AU combinations that can theoretically be produced based on the set of rules given, and stores it in a list named `subsets`.

GENERATING

```
#Cat AUs
AUs = [5, 143, 145, 47, 48, 68, 69, 109, 110, 12, 116, 17, 118, 25, 26, 27, 19, 37, 137, 190, 200, 201, 202, 101, 102, 103, 104, 105, 106, 107]
word_bank = sorted(AUs)
len(word_bank)
print(word_bank)
```

```
[5, 12, 17, 19, 25, 26, 27, 37, 47, 48, 68, 69, 101, 102, 103, 104, 105, 106, 107, 109, 110, 116, 118, 137, 143, 145, 190, 200, 201, 202]
```

```
#keeping track of how long it takes to run
total_start = time.time()
start = time.time()
```

(This below function is the one that takes the bulk of the time, with 35.47 minutes.)

```
subsets = []
subsets = generate_combinatorial_subsets(word_bank, rules)
```

```
end = time.time()
print(f"Time taken: {end - start:.2f} seconds ({(end - start)/60:.2f} minutes)")
```

```
Time taken: 1201.36 seconds (20.02 minutes)
```

```
print('The function has calculated that there are: ' + str(len(subsets)) + " possible combinations")
```

```
The function has calculated that there are: 1062719 possible combinations
```

The following snippet of code turns elements in the `subsets` list into a concatenated string.

From `(15, 20, 22, 26, 28, 31)` in subsets to `'15+20+22+26+28+31'` in `concatenated_data`.

```
start = time.time()
```

```
concatenated_data = string_format(subsets)
```

```
end = time.time()
print(f"Time taken: {end - start:.2f} seconds")
```

```
Time taken: 1.06 seconds
```

```
combos = pd.DataFrame(concatenated_data)
combos.columns = ['combinations']
```

PART 3: Comparing

Now that our possible generated combos are in a data frame named `combos`, and our list of already observed AUs is in a data frame named `observed`, we can now continue to look at how many of the possible combinations we have actually seen in observations of cats.

Checking out the structure of `observed` and `combos`:

```
observed.head()
```

	given_AUs
0	47
1	101
2	102
3	103
4	104

```
combos.head()
```

	combinations
0	5
1	12
2	17
3	25
4	26

The following block of code checks matches between `combos` and `observed`. If there's a match, it removes it from the `combos` (generated subsets) list. This way, as it removes all the seen combination, we are left with a remaining list of all the combos that have NOT been observed in the wild yet.

After checking equivalence, we break from the `if` check because there is no need to check a certain combination with all the rest of the combinations when its match has already been found.

```
start = time.time()
```

```
unseen = []
seen = []

# Create a copy of combos['combinations'] to work with so we don't modify the original
remaining_combos = combos['combinations'].copy()
remaining_combos = remaining_combos.tolist()

# Comparing and dividing into list of seen and unseen AUs
for x in combos['combinations']:
    match_found = False
    for y in observed["given_AUs"]:
        if x == y:
            #print("\nPossible combo: " + str(x) + " is present in the list of observed AUs. A match with: ", str(y))
            seen.append(x)
            remaining_combos.remove(x)
            match_found = True
            break
```

```
end = time.time()
print(f"Time taken: {end - start:.2f} seconds")
```

```
Time taken: 25.51 seconds
```

We will now check for observations that have been flagged as not having a match in the generated combinations subsets, so that we may investigate them. They will be stored in a list named `observed_but_not_possible`.

```
observed_combinations = observed["given_AUs"].tolist()
generated_combinations = combos['combinations'].tolist()

observed_but_not_possible = []

for obs in observed_combinations:
    if obs not in generated_combinations:
        observed_but_not_possible.append(obs)

print("Number of observed combinations not found in generated combos:", len(observed_but_not_possible))
```

```
Number of observed combinations not found in generated combos: 3
```

```
observed_but_not_possible
```

```
['47+104+106', '37+104+105+143+201', '12+25+26+47+104+106+116']
```

```
len(remaining_combos)
```

```
1062447
```

```
unseen = remaining_combos
```

```
print("length of total generated subsets (combos): " + str(len(combos)))
print("length of observed combos: " + str(len(observed)))
print("\nunseen: " + str(len(unseen)))
print("seen: " + str(len(seen)))
print("observed combos that \"don't exist\": " + str(len(observed_but_not_possible)))
```

```
length of total generated subsets (combos): 1062719
length of observed combos: 275

unseen: 1062447
seen: 272
observed combos that "don't exist": 3
```

```
print("Percentage of seen combos out of all possible combos: " + str(round(len(seen) / len(combos) * 100, 2)) + "%")
print("(This is " + str(len(seen)) +" out of " + str(len(combos)) + ")")

print("\nThe first 5 combos seen are: ")
print(seen[0:5])

print("\nThe first 10 combos not seen are: ")
print(unseen[0:10])
```

```
Percentage of seen combos out of all possible combos: 0.03%
(This is 272 out of 1062719)

The first 5 combos seen are:
['47', '101', '102', '103', '104']

The first 10 combos not seen are:
['5', '12', '17', '25', '26', '48', '68', '69', '107', '118']
```

Next, each list is turned into a data frame so it can be exported.

```
seen_df = pd.DataFrame(seen, columns=['seen_combos'])
seen_df.to_excel('Cats-seen_combos.xlsx', index=False)
```

The unseen list is too large to directly export to Excel in one sheet...

```
ValueError: This sheet is too large! Your sheet size is: 1,062,719, 1 Max sheet size is: 1,048,576, 16384
```

...So we will split it into two sheets. Thank you to my advisor Dr. Marcus Birkenkrahe for showing me this technique.

```
unseen_df = pd.DataFrame(unseen, columns=['unseen_combos'])
```

```
import sys

size_in_mb = sys.getsizeof(unseen_df) / 1e6
print(f"Size of list in MB: {size_in_mb:.2f}")

max_rows = 1048570  # Sets the maximum number of rows per chunk
num_chunks = (len(unseen_df) + max_rows - 1) // max_rows  # Calculate the number of chunks

for i in range(num_chunks):
    # Compute chunk start and end index
    start_index = i * max_rows
    end_index = min((i + 1) * max_rows, len(unseen_df))

    # Extract chunk from the list
    chunk = unseen_df[start_index:end_index]

    # Convert chunk to DataFrame
    df = pd.DataFrame(chunk, columns=["unseen_combos"])


    # Save to Excel: each chunk in a separate sheet or file
    file_name = f"Cats-unseen_combos-part{i + 1}.xlsx"
    df.to_excel(file_name, index=False)
```

```
Size of list in MB: 97.52
```

```
observed_but_not_possible_df = pd.DataFrame(observed_but_not_possible, columns=['observed_but_not_in_subsets'])
observed_but_not_possible_df.to_excel('Cats-flagged.xlsx', index=False)
```

```
total_end = time.time()
print(f"Time taken: {total_end - total_start:.2f} seconds ({(total_end - total_start)/60:.2f} minutes)")
```

```
Time taken: 1250.42 seconds (20.84 minutes)
```

PART 4 - Databases! :)

The next steps upload the generated subsets (`combos`), observed AUs (`observed`), `seen`, and `unseen` into separate SQLite3 databases so they may be queried.

```
import sqlite3
```

COMBOS database

```
db_name = 'generated_combos.db'
conn = sqlite3.connect(db_name)

table_name = 'all_possible_subsets'
combos.to_sql(table_name, conn, index=False, if_exists='replace')
```

```
1062719
```

```
query = "SELECT * FROM all_possible_subsets WHERE combinations LIKE '%10%'"
possibles = pd.read_sql_query(query, conn)
print(possibles)
```

```
                                           combinations
0                                                   101
1                                                   102
2                                                   103
3                                                   104
4                                                   105
...                                                 ...
1040575  25+27+48+69+102+103+104+109+110+116+118+137+14...
1040576  25+27+48+69+103+104+105+109+110+116+118+137+14...
1040577  25+27+48+69+103+104+105+109+110+116+118+137+14...
1040578  25+27+48+69+103+104+105+109+110+116+118+137+14...
1040579  25+27+48+69+103+104+105+109+110+116+118+137+14...

[1040580 rows x 1 columns]
```

OBSERVED AU database

```
db_name = 'observed_data.db'
conn = sqlite3.connect(db_name)

table_name = 'observed_AUs'
observed.to_sql(table_name, conn, index=False, if_exists='replace')
```

```
275
```

```
query = "SELECT * FROM observed_AUs LIMIT 10"
observeds = pd.read_sql_query(query, conn)
print(observeds)
```

```
  given_AUs
0        47
1       101
2       102
3       103
4       104
5       105
6       106
7      5+68
8      5+69
9     5+101
```

SEEN database

```
db_name = 'seen_combos.db'
conn = sqlite3.connect(db_name)

table_name = 'seen'
seen_df.to_sql(table_name, conn, index=False, if_exists='replace')
```

```
272
```

```
query = "SELECT * FROM seen LIMIT 10"
seen_sql = pd.read_sql_query(query, conn)
print(seen_sql)
```

```
  seen_combos
0          47
1         101
2         102
3         103
4         104
5         105
6         106
7        5+68
8        5+69
9       5+101
```

UNSEEN database

```
db_name = 'unseen_combos.db'
conn = sqlite3.connect(db_name)

table_name = 'unseen'
unseen_df.to_sql(table_name, conn, index=False, if_exists='replace')
```

```
1062447
```

```
query = "SELECT * FROM unseen LIMIT 5"
unseen_top_5 = pd.read_sql_query(query, conn)
print(unseen_top_5)
```

```
  unseen_combos
0             5
1            12
2            17
3            25
4            26
```

```
query = "SELECT * FROM unseen WHERE unseen_combos LIKE '%5%' AND unseen_combos LIKE '%15%'"
both_5_and_15 = pd.read_sql_query(query, conn)
print(both_5_and_15)
```

```
Empty DataFrame
Columns: [unseen_combos]
Index: []
```

Results

Refer to *Using Facial Action Coding Systems and Data Engineering to Evaluate the Communicative Potential in Mammals* (Mahmoud, et al.) to read about the results.

```
print("Cats\n")
print("length of total generated subsets: " + str(len(combos)))
print("length of observed combos: " + str(len(observed)))
print("\nunobserved: " + str(len(unseen)))
print("observed: " + str(len(seen)))
print("flagged combos: " + str(len(observed_but_not_possible)))
```

```
Cats

length of total generated subsets: 1062719
length of observed combos: 275

unobserved: 1062447
observed: 272
flagged combos: 3
```

References:

Birkenkrahe, Marcus. Saving large data sets to Excel. Gist. Retrieved February 9, 2024, from https://gist.github.com/birkenkrahe/57142fc39cf7b174bee597825126c83f

Caeiro, C. C., Burrows, A. M. & Waller, B. M. Development and application of CatFACS: Are human cat adopters influenced by cat facial expressions? Applied Animal Behaviour Science 189, 66-78 (2017). https://doi.org:https://doi.org/10.1016/j.applanim.2017.01.005

Scott, L., Florkiewicz, B. N. Feline faces: Unraveling the social function of domestic cat facial signals. Behavioural Processes 213, 104959 (2023). https://doi.org:https://doi.org/10.1016/j.beproc.2023.104959
