# Supplementary material for: Examining Mammalian facial behavior using Facial Action Coding Systems (FACS) and combinatorics
Source: PLoS One. 2025 Jan 27;20(1):e0314896. doi: 10.1371/journal.pone.0314896 (PMC11771922; doi:10.1371/journal.pone.0314896)
Supplement: Attachment — Submitted filename: Mahmoud et al._Comments Addressed.docx [file pone.0314896.s006.docx]

**Reviewers' Comments Addressed**

Dear Dr. Komiyama,

Thank you for your careful consideration of our manuscript. We thank you and the three anonymous reviewers for the constructive comments and suggestions on the manuscript. We recognize that it has been difficult to find reviewers at this time, and we are very thankful for your time and effort.

Below, you will find a list of reviewer comments and corresponding revisions made to the manuscript (in blue, with line numbers included). All changes listed in this document have been track changed in the manuscript. A clean version of the manuscript is provided for comparison. After making these revisions, we hope the manuscript is suitable for publication in PLOS One.

We look forward to hearing from you.

Sincerely,

Brittany Florkiewicz, Ph.D.

Department of Psychology

Lyon College

On behalf of all authors.

**Reviewer #1**

While I agree that there still is a lot of work to be done when studying facial movements across species and that improvements are needed in how FACS is used in animal behavior / comparative psychology, I am not convinced that the authors really achieve what they set out to do in this study. It is possible that additional explanations and methodological details can be added to make it clearer for the reader what the contribution of the paper is. However, as it stands, I am struggling to understand the value of comparing the proportion of observed facial configurations with the total number of possible facial configurations. Other approaches, relying on evaluating observation effort based on the cumulative frequency of facial combinations over time seem more straightforward (e.g., Roberts et al., 2019) and achieve the same goal.

In addition, and importantly, to be able to address questions relating to communication, it is crucial to ascertain that behaviour (facial or otherwise) is indeed communicative. It is highly unlikely that all the possible combinations of AUs/ADs have communicative value (as signals or cues). While it is possible that considering these hypothetical, possible facial configurations is indeed interesting, but I believe that this needs to be discussed more clearly and more thoroughly throughout the paper.

We express our gratitude to reviewer #1 for their valuable feedback on our paper. We have carefully considered these points and provided detailed responses below.

**Detailed comments:**

4. Given the structure of the previous sentence, this should be “One such approach is…” or “Such approaches include…”.

We have revised the sentence so that it starts with “such approaches include” (line 4).

6-7 I don’t think this is a fair representation of the work that has been done with FACS. It neglects comparisons between species with regards to social structure (e.g., Scheider et al., 2014), social tolerance (e.g., Rincon et al., 2023), as well as studies investigating the perception of facial movements (e.g., Parr et al., 2008). Also, given that FACS was developed to document facial movements, it can hardly be used for something other than studying communication via the face.

We have revised the sentence as follows: “Some studies have utilized FACS to analyze facial signaling, recording the quantity of morphologically distinct facial signals a species can generate” (lines 6-7).

10-13 This read more like an overview of the methods rather than the aims of the study. I guess the aim could be to evaluate how representative the current literature is in documenting the full extent of facial behaviour in these species, or something along those lines.

We have revised the sentence as follows: “Our study aimed to assess how well the existing literature represents the potential range of facial signals in two previously studied species: chimpanzees (Pan troglodytes) and domesticated cats (Felis silvestris catus).” (lines 12-14). We have also implemented this change at the start of our “Current Study” section.

13 I would call them guidelines rather than rules.

We have changed the word ‘rules’ to ‘guidelines’ in the abstract and throughout the paper.

30 Here and throughout the paper, in text citations contain a lot of unnecessary initials. This is likely due to how these papers were added in your reference manager (e.g., naming the author C.C. Caeiro in one case and Catia C. Caeiro in another will lead to citations including initials as the software considers they are different authors).

We have reviewed the paper and manually edited in-text citations to ensure consistency in citation style.

35 The reference to Florkiewicz (2023) here and in other places (e.g., lines 430 and 431) is not appropriate. Here I would cite Waller et al. (2020) here. Over reliance on self-citation is not good practice. Obviously previous works of the senior author are highly relevant when discussing facial movements in several species, including chimpanzees and cats. However, they are not suitable when making broader statements regarding their socio-ecology. Florkiewicz (2023) also appears to be missing in the reference list.

We have removed the citation for Florkiewicz et al. 2023 and made other citation cuts throughout the paper accordingly. Additionally, we added Florkiewicz et al. 2023 to the reference list.

51-52 These studies go beyond documenting the facial repertoire and test hypotheses relating to the social use of facial movements (Scheider et al., 2016) and species differences relating to social structure (Scheider et al., 2014).

We have added the following sentence follow to clarify the aim of these studies: “It is important to note that these studies documented facial signaling repertoires mainly to test hypotheses related to the social use of facial movements (Scheider et al., 2016; Florkiewicz et al., 2018) and to assess differences across species with varying social structures and organizations (Scheider et al., 2014; Florkiewicz et al., 2023)” (lines 56-59).

62 I think it is important to distinguish between observed facial configurations and facial signals. Definitions of a signal varies but they are often quite stringent. For example, Laidre and Johnstone (2013) define signals as “(1) acts or structures produced by signalers, which (2) evolved for the purpose of conveying information to recipients, such that (3) the information elicits a response in recipients, and (4) the response results in fitness consequences that, on average, are positive for both the signaler and the recipient”. Unless the configurations are observed in a communicative context, potentially leading to a response from the receiver, I'm not convinced the number of possible configurations is particularly meaningful.

This is a valid point. It is possible that not all generated AU combinations are communicative. For this reason, we have switched from using the phrase “signal/cue” to “configurations” throughout the manuscript when referring to our generated lists.

We have also added the following sentence for (at the beginning of the “Current Study” section) for clarification: “Although we may not be able to ascertain the communicative function of every facial configuration without additional research, compiling a thorough list of all possible facial configurations can still help in assessing the facial mobility and communication potential in mammals. This information can also be utilized to guide future data collection and coding efforts for each species.” (lines 148-152).

66 Here again, a broader review of the literature would be beneficial (e.g., Rincon et al., 2023; Scheider et al., 2014).

Thank you for bringing the Rincon et al. study to our attention! This is a great study. We have added information regarding both Rincon et al. and Scheider et al. 2014 in the following sections:

“…compare the complexity of communication among macaque species with varying degrees of social tolerance (Rincon et al., 2023),…” (lines 48-49).

“In hylobatids and domesticated cats, there are distinct facial signals (identified with FACS) that are exclusive to affiliative contexts, indicating variations in the social function and use of these muscle movement combinations (Scheider et al., 2014; Scott & Florkiewicz et al., 2023).” (lines 70-73).

“It is important to note that these studies documented facial signaling repertoires mainly to test hypotheses related to the social use of facial movements (Scheider et al., 2016; Florkiewicz et al., 2018) and to assess differences across species with varying social structures and organizations (Scheider et al., 2014; Florkiewicz et al., 2023)” (lines 56-59).

84-86 Intuitively, this makes sense, however the example suggests the opposite, since there were more individuals hylobatids, but fewer movements observed.

We have added the following sentence to address this possibility: “Collecting and coding additional video footage can be time-consuming, potentially limiting research opportunities and insights into the communication behaviors of domesticated cats. For this reason, it is crucial to have a method for assessing whether additional data collection and coding efforts will yield new facial signals or if a limit has been reached.” (lines 105-109).

106 Overall, the section “Current Study” is a bit messy, including sections relating to methods, aims and rationale. Maybe this can be reorganised in a clearer, more coherent way.

We have removed repetitive sections (second paragraph) and moved sections related to the future utility of our configuration lists (last paragraph) to the discussion section.

124-131 This goes some way in addressing my previous comment regarding the importance of distinguishing between communicative and other movements. However, I'm not sure it is very convincing. I also think it should come earlier and be better integrated in the introduction. A possible solution is to avoid the term signal/cue and talk about facial configurations. In the discussion, whether these configurations are communicative or not can be addressed.

We have switched from using the phrase “signal/cue” to “configurations” throughout the manuscript when referring to our generated lists. We also mention the limitations of our study at the beginning of our “Current Study” section: “Although we may not be able to ascertain the communicative function of every facial configuration without additional research, compiling a thorough list of all possible facial configurations can still help in assessing the facial mobility and communication potential in mammals. This information can also be utilized to guide future data collection and coding efforts for each species.” (lines 148-152).

Finally, we address the need for future studies to assess the communicative function (or lack thereof) of unobserved facial configurations: “It is important to note that in order to determine whether a facial configuration is communicative, the behavioral responses of recipients must be evaluated. If signals help to elicit behavioral responses in recipients that have positive fitness consequences for both signalers and recipients (Laidre & Johnstone, 2013), then there should be observable behavioral responses from recipients. This may also involve a clear attempt to elicit a behavioral response from the signaler's perspective, with evidence of intentionality and/or goal-directed behavior (Scheider et al., 2014; Byrne et al., 2017). It is only by coding the behavioral responses of both signalers and recipients that we can determine which configurations generated by our Python models are communicative or non-communicative. Increasing sample sizes and hours of video footage may reveal additional combinations that are used as facial signals, but it is also possible that our “observed” datasets represent the full communicative potential of each species.” (lines 376-386).

203 Please explain how you determined whether facial movements were used to communicate.

We have added the following information to our manuscript: “In both studies, facial signals are defined as facial muscle movements performed by a signaler when communicating with recipients to elicit a behavioral response (Florkiewicz et al., 2023; Scott & Florkiewicz et al., 2023). Like other types of communicative signals, facial signals evolved to convey information from signals to recipients and the recipient’s behavioral response has positive fitness consequences for both signalers and recipients (Laidre & Johnstone, 2013). For both studies, facial signals that led to a behavioral response or indicated a desired behavioral response were coded.” (lines 197-204).

207-209 Please explain how you determined the apex of the facial configuration.

We have added the following paragraph to our methods section for clarification: “Facial signals were coded at their apex, or the peak production, when all facial muscle movements involved in the signaling bout were present and at their highest intensity. For example, a mammal may initially produce a facial signal of low intensity, involving only 2 facial muscle movements. A couple of seconds later, an additional 3 muscle movements might be added during the facial signaling bout. It is when all 5 facial muscle movements are present and produced that the apex is reached, and this is when we code the facial signal using FACS” (lines 207-213).

233 A full stop and space are missing before “We”.

Thank you for catching this! We have added the stop and space before “We”.

304-306 I'm not sure I understand this. Do you mean you calculated the proportion of observed combinations based on the possible number of combinations? I think more details are required in this section.

We have modified this sentence to state the following: “Specifically, we compared the proportion of facial muscle movements observed across all facial configurations in our “observed” lists for each species (by taking the raw counts for each facial muscle movement and dividing them by the total number of observed facial configurations) to proportions of facial muscle movements generated by our combinatorial models (i.e., our “possible” configuration lists)” (lines 283-285). We have also modified the captions of Table 1 and Table 2 to describe this procedure.

336-339 This seems more appropriate in the discussion section. I believe that a coding error is also a possible explanation. Also, please explain what you mean by blended effect.
344-349 I think that instead of selecting a few AUs/ADs to illustrate the results, it would be better to just refer to the table. This would also avoid duplicate information in the text and in the table.

We have addressed the possibility of manual coding error to this section: “First, some of these facial signals may have been produced on only one side of the face, resulting in asymmetry. The second possibility is that there could have been multiple signal peaks due to blended effects. This means that multiple types of communicative facial configurations are rapidly combined together, making it difficult to discern which apex to focus on for coding. Finally, these flagged facial signals may be the result of manual coding errors. The three facial signals that were flagged by our cat combinatorial model appear to reflect coding errors associated with visibility issues. In two cases, there appeared to be difficulty distinguishing between ear rotating and narrowing movements (EAD104 versus EAD106). In the last case, there was no direct observation of lip parting movement (AU25), but evidence of tongue protrusion (AD37) was present” (lines 467-476We also moved this information to the discussion section to help address the comment related to lines 468-470 (below).

We have omitted the in-text examples for select AUs/ADs for both the chimpanzee and cat results sections.

350-355 I think the table title needs to be revised to increase clarity (for example, adding the label in plain English for each AU/AD (e.g., AU6 cheek raiser)) and/or more details can be added in the text in the methods. As I understand the number observed is the number of combinations (which you call signals/cues) observed including this AU/AD (11 for AU6). The prop observed is this number divided by the total number of unique combinations observed (57). I’m not too sure about what this comparison tells us and why it is important.

We have revised the caption of Table 1 for clarity: “Table 1. A list of all facial muscle movements mentioned in the chimpFACS manual (Vick et al., 2007) and considered in our present study. We include the raw count and proportion of facial muscle movements identified in previous studies (i.e., our “observed” configuration list), as well as the raw count and proportion of facial muscle movements that were in our “unobserved” configuration list (identified using our combinatorial model). The proportion of "observed" facial muscle movements was calculated by dividing the number of instances that each facial muscle movement was observed (in column 3) by the total number of facial configurations in our "observed" list (N=57). The proportion of “unobserved” facial muscle movements was calculated by dividing the number of instances that each facial muscle was generated by our Python model (in column 5) by the total number of facial configurations in our “unobserved” list (N=238,022). The last column displays the results (p-values) of our two-proportion z-test for each facial muscle movement” (lines 319-330)).

These changes were also applied to the caption of Table 2, which mirrors the sentence structure of Table 1.

Additionally, we have added a new column (2) that includes the full description of each AU/AD.

355 Given the number of tests and the probable occurrence of pseudoreplication, I am not sure that so many z-tests are appropriate. A qualitative comparison is probably more suitable and sufficient.

We have excluded the z-tests from our manuscript and instead, we have concentrated on qualitative comparisons only. In Tables 1 & 2, we have switched the last column to include information about differences in proportion between facial muscle movements in our observed and unobserved configuration lists.

357 The same comments made for the chimpanzee section apply to the cat section. There are repetitions between the text and the table. The table could be clearer, and you should consider dropping the tests for more qualitative comparisons.

We have omitted the in-text examples for select AUs/ADs for both the chimpanzee and cat results sections. We have also modified the Table 2 caption (see comment above) and added a column (2) that includes the full description of each AU/AD.

392-395 This is potentially useful but please explain how your approach can inform researchers’ decision to invest more time and resources when studying facial behaviour and how it can improve the accuracy of FACS coding. This is not clear to me, even after reading the whole discussion.

We have moved this information to later parts of the introduction so that we address & clarify each point separately. See lines (x-x) for information regarding coding efforts and lines x-x regarding coding accuracy.

**CODING EFFORTS:** “By comparing previously observed facial signals with unobserved facial configurations generated through combinatorial approaches, we can help researchers decide whether to allocate additional time and resources to study the facial signaling repertoires of chimpanzees and domesticated cats in the future. Researchers who invest a lot of time and resources in collecting and coding facial signaling data using FACS may face high risks and limited rewards if they are solely focused on documenting facial signaling repertoires. This is especially true if our "observed" configuration list represents the full communicative potential of each species. For instance, previous studies have estimated that coding 1 minute of video footage with FACS takes approximately 1-2 hours (Bartlett et al., 2003; Donato et al., 2010). This estimate does not account for the roughly 100 hours needed to attain proficiency and certification in using FACS (Bartlett et al., 2003; Donato et al., 2010) or the amount of time needed to collect video footage for coding. But future studies do not have to solely focus on documenting each species' communicative potential. Instead, their findings can be indirectly used to achieve this purpose. There are various research questions that FACS-based approaches can address, such as detecting instances of rapid facial mimicry (Davila-Ross & Palagi, 2022) or documenting the effects of multimodal communication on facial signaling form and function (Oña et al., 2019). Information on facial signaling repertoires can be used to detect instances of rapid facial mimicry and assess the role of multimodal communication, while also contributing to a comprehensive list of communicative facial configurations for chimpanzees and domesticated cats.” (lines 387-405).

**CODING ACCURACY:** “Our example of potential underreporting of AD19 also leads us to one of the strengths of our study: our generated lists of facial configurations can improve coding accuracy. Our configurations lists can highlight underreported facial muscle movements and also evaluate combinations of movements according to FACS guidelines. Our combinatorial model in Python was able to detect 57 out of the 66 morphologically distinct facial signals found in our “observed” configuration list for chimpanzees. Similarly, it detected 272 out of the 275 morphologically distinct facial signals found in “observed” configuration list for domesticated cats. There are three possible explanations as to why our combinatorial model flagged 9 chimpanzee facial signals in our “observed” configuration list. First, some of these facial signals may have been produced on only one side of the face, resulting in asymmetry. The second possibility is that there could have been multiple signal peaks due to blended effects. This means that multiple types of communicative facial configurations are rapidly combined together, making it difficult to discern which apex to focus on for coding. Finally, these flagged facial signals may be the result of manual coding errors. The three facial signals that were flagged by our cat combinatorial model appear to reflect coding errors associated with visibility issues. In two cases, there appeared to be difficulty distinguishing between ear rotating and narrowing movements (EAD104 versus EAD106). In the last case, there was no direct observation of lip parting movement (AU25), but evidence of tongue protrusion (AD37) was present.

Our combinatorial models still reveal a high level of accuracy for our two previously published FACS-coded datasets. Most of the facial configurations observed in our lists for chimpanzees (86.364%) and domesticated cats (98.910%) were not flagged, meaning that they conformed to the FACS guidelines. Manual coders can maintain a high level of accuracy by frequently referencing the FACS for guidance and renewing certifications when significant time has passed. However, these techniques may not be as easily applied when coding automation is being used. In recent years, there has been rapid development of automated facial muscle movement detection systems, most notably using landmark features (Wu & Ji, 2019). We suggest utilizing these automated techniques in conjunction with our combinatorial models to provide an additional level of assistance in accurately identifying facial muscle movements during communication. Many of these automated approaches have been developed with inspiration from FACS, making our combinatorial approaches compatible. For example, landmark detection systems for domesticated cats have been developed using inspiration from FACS-based coding techniques (Feighelstein et al., 2022; Finka et al., 2019; Martvel, Farhat, et al., 2023; Martvel, Shimshoni, et al., 2023). These landmark detection systems achieve a high level of accuracy when using high-quality video footage gathered in controlled environments (Feighelstein et al., 2022). Since a large portion of animalFACS studies are conducted in captive environments (including those with chimpanzees and domesticated cats; Bennett et al., 2017; Correia-Caeiro et al., 2017; Florkiewicz et al., 2023; Parr et al., 2008; Scott & Florkiewicz et al., 2023; Waller et al., 2013; Waller et al., 2016), these landmark detection techniques can be highly beneficial and dependable. Configuration data generated from our combinatorial models can be used to assess the accuracy of landmark detection outputs by determining the feasible combinations of facial muscle movements” (lines 459-499).

401 I’m not sure this is a valid statement. I’m no expert but I believe chimpanzees are one of the widely studied species with FACS.

We have added the following sentence to address this point: “Although chimpanzees and domesticated cats are widely studied with FACS, there are few studies that publish comprehensive lists of facial signals” (lines 372-374).

405-406 Deciding whether facial configurations are communicative, or whether they are cues or signals is only possible by considering the behaviour of the receiver. This is a limitation of the approach presented here and it should be discussed thoroughly.

We have addressed this by adding the following information to our discussion section: “It is important to note that in order to determine whether a facial configuration is communicative, the behavioral responses of recipients must be evaluated. If signals help to elicit behavioral responses in recipients that have positive fitness consequences for both signalers and recipients (Laidre & Johnstone, 2013), then there should be observable behavioral responses from recipients. This may also involve a clear attempt to elicit a behavioral response from the signaler's perspective, with evidence of intentionality and/or goal-directed behavior (Scheider et al., 2014; Byrne et al., 2017). It is only by coding the behavioral responses of both signalers and recipients that we can determine which configurations generated by our Python models are communicative or non-communicative” (lines 376-384).

413-415 This example needs more details to be clear and convincing.

We have clarified this section by adding information about future studies that could help to generate lists of observed facial configurations: “But future studies do not have to solely focus on documenting each species' communicative potential. Instead, their findings can be indirectly used to achieve this purpose. There are various research questions that FACS-based approaches can address, such as detecting instances of rapid facial mimicry (Davila-Ross & Palagi, 2022) or documenting the effects of multimodal communication on facial signaling form and function (Oña et al., 2019). Information on facial signaling repertoires can be used to detect instances of rapid facial mimicry and assess the role of multimodal communication, while also contributing to a comprehensive list of communicative facial configurations for chimpanzees and domesticated cats” (lines 398-405).

419-420 This is a mathematical relationship, not a finding of this study. The more component in a system, the more possible combinations.

We have rephrased this sentence as follows: “Because there are more documented AUs/ADs in the catFACS manual compared to the chimpFACS manual, our model generated a greater number of facial configurations for cats” (lines 406-408).

420-424 I would be a bit more cautious when making such statements since there is limited work available and what would be important would be to assess the communicative value of these movements.

We reworked this section to take a more conservative approach while also addressing the need for future studies: “Because there are more documented AUs/ADs in the catFACS manual compared to the chimpFACS manual, our model generated a greater number of facial configurations for domesticated cats. Domesticated cats, for example, exhibit a wide range of ear movements that have not been described in the chimpFACS (Vick et al., 2007). Furthermore, additional movements associated with the pupils, whiskers, and tongue have been documented in the catFACS (Correia-Caeiro et al., 2017) but not in the chimpFACS (Vick et al., 2007). However, our estimates regarding the number of possible facial configurations for each species may change when including additional facial action descriptors (such eye movements, head positions, and head movements). When these additional facial muscle movements and descriptors are considered, the number of morphologically distinct facial signals in chimpanzees, for example, increases from 66 to 357 (Florkiewicz et al., 2023). However, future studies should assess whether these additional facial action descriptors are communicative before considering their inclusion into the observed list of facial configurations” (lines 406-418).

430-431 The references to Florkiewicz (2023) are not appropriate here. Please go through the entire manuscript and ensure self-citations are appropriate. Also make sure this reference is included in the reference list.

We have replaced the Florkiewicz (2023) references with references that relate to the social organization of both species. We have also reviewed the manuscript and removed any inappropriate citations of this paper. Finally, we have added it to the reference list at the end.

444 A major issue with the proposed methodology is that it is not clear what these proportions mean and what a difference between proportion observed and unobserved tells us.

We have clarified our rationale for calculating and comparing proportions by adding the following information: “Comparing proportions of facial muscle movements can help identify whether some facial muscle movements are at greater risk of being overlooked during the coding process, which may lead to underreporting. We have used our unobserved configuration lists as a reference point, as they may represent the full communicative potential of each species (although additional research is needed to verify this idea). In our analysis, we found that some facial muscle movements were identified at similar rates in our “observed” and “unobserved” configuration lists. Examples include AUs/ADs that have less than a 1% difference in documented proportions, such as AU17 (chin raiser) and AU26 (jaw drop) in chimpanzees and AU26 (jaw drop) and EAD101 (ears forward) in domesticated cats. However, many of the facial muscle movements included in our study appear more frequently in our unobserved configuration lists than in our observed configuration lists, which could suggest underreporting” (lines 429-440).

453-455 A above, explaining what these differences mean or could mean is crucial to explain the possible value of the approach presented in the manuscript.

We have clarified some of the wording in this paragraph to help address what these differences mean: “In addition to comparing the number of previously documented facial configurations to the facial configurations generated through our combinatorial models, we also examined the proportion of discrete facial muscle movements observed across configurations. Comparing proportions of facial muscle movements can help identify whether some facial muscle movements are at greater risk of being overlooked during the coding process, which may lead to underreporting. We have used our unobserved configuration lists as a reference point, as they may represent the full communicative potential of each species (although additional research is needed to verify this idea). In our analysis, we found that some facial muscle movements were identified at similar rates in our “observed” and “unobserved” configuration lists. Examples include AUs/ADs that have less than a 1% difference in documented proportions, such as AU17 (chin raiser) and AU26 (jaw drop) in chimpanzees and AU26 (jaw drop) and EAD101 (ears forward) in domesticated cats. However, many of the facial muscle movements included in our study appear more frequently in our unobserved configuration lists than in our observed configuration lists, which could suggest underreporting.” (lines 427-440).

461-463 This is a clearer outcome, but technically, it is achievable by just following the FACS manual guidelines.

We have acknowledged this possibility and clarified the importance of our study by adding some new information and restructuring one of our previous paragraphs: “Our combinatorial models still reveal a high level of accuracy for our two previously published FACS-coded datasets. Most of the facial configurations observed in our lists for chimpanzees (86.364%) and domesticated cats (98.910%) were not flagged, meaning that they conformed to the FACS guidelines. Manual coders can maintain a high level of accuracy by frequently referencing the FACS for guidance and renewing certifications when significant time has passed. However, these techniques may not be as easily applied when coding automation is being used. In recent years, there has been rapid development of automated facial muscle movement detection systems, most notably using landmark features (Wu & Ji, 2019). We suggest utilizing these automated techniques in conjunction with our combinatorial models to provide an additional level of assistance in accurately identifying facial muscle movements during communication. Many of these automated approaches have been developed with inspiration from FACS, making our combinatorial approaches compatible. For example, landmark detection systems for domesticated cats have been developed using inspiration from FACS-based coding techniques (Feighelstein et al., 2022; Finka et al., 2019; Martvel, Farhat, et al., 2023; Martvel, Shimshoni, et al., 2023). These landmark detection systems achieve a high level of accuracy when using high-quality video footage gathered in controlled environments (Feighelstein et al., 2022). Since a large portion of animalFACS studies are conducted in captive environments (including those with chimpanzees and domesticated cats; Bennett et al., 2017; Correia-Caeiro et al., 2017; Florkiewicz et al., 2023; Parr et al., 2008; Scott & Florkiewicz et al., 2023; Waller et al., 2013; Waller et al., 2016), these landmark detection techniques can be highly beneficial and dependable. Configuration data generated from our combinatorial models can be used to assess the accuracy of landmark detection outputs by determining the feasible combinations of facial muscle movements” (lines 477-499).

465-468 It is quite repetitive to present the same information in a different form in different sentences. The information could be presented more concisely.

We have omitted the second sentence to minimize repetition.

468-470 This is not clear. it needs to be explained further. Please explain why or how asymmetrical movements affect the occurrence of theoretically impossible combinations. Similarly, please explain what graded effects are and why or how they can affect the occurrence of theoretically impossible combinations.

We have clarified this statement by relocating information from the results section and adding new information: “Our example of potential underreporting of AD19 also leads us to one of the strengths of our study: our generated lists of facial configurations can improve coding accuracy. Our configurations lists can highlight underreported facial muscle movements and also evaluate combinations of movements according to FACS guidelines. Our combinatorial model in Python was able to detect 57 out of the 66 morphologically distinct facial signals found in our “observed” configuration list for chimpanzees. Similarly, it detected 272 out of the 275 morphologically distinct facial signals found in “observed” configuration list for domesticated cats. There are three possible explanations as to why our combinatorial model flagged 9 chimpanzee facial signals in our “observed” configuration list. First, some of these facial signals may have been produced on only one side of the face, resulting in asymmetry. The second possibility is that there could have been multiple signal peaks due to blended effects. This means that multiple types of communicative facial configurations are rapidly combined together, making it difficult to discern which apex to focus on for coding. Finally, these flagged facial signals may be the result of manual coding errors. The three facial signals that were flagged by our cat combinatorial model appear to reflect coding errors associated with visibility issues. In two cases, there appeared to be difficulty distinguishing between ear rotating and narrowing movements (EAD104 versus EAD106). In the last case, there was no direct observation of lip parting movement (AU25), but evidence of tongue protrusion (AD37) was present” (lines 459-476).

**Reviewer #2**

This manuscript aims to examine mammalian facial behavior by means of FACTS. This manuscript does not present a novel idea. It is not written in a regular journal paper format. I recommend to read a peer journal paper how to write, particularly, the method and results sections. Solely explaining the programming code is not for scientific journal papers.

Thank you for your feedback on our article. We have significantly reduced the content in the methods section to improve clarity. Additionally, we have revised the tables in our results section and removed specific details about AUs/ADs to avoid duplication between the text and tables.

**Reviewer #3**

I have a few comments pertaining to the clarity of the manuscript, justification of methods, implications of these findings, and the application of this method. My most substantial comments regard the fact that the manuscript is framed around the potential for studies to underestimate repertoire richness without doing any correction for this in their analyses. Below I suggest a potential additional analysis which would round out the argument and complement the results presented.

**Major comments:**

Line 81: I think the authors raise a valid point that sampling effort could very well influence the number of signals observed. This is similar to what one observes in sampling species diversity in a given ecosystem, in which increased sampling effort increases the number of species found up until a point in which the number of new species found for each man hour begin to decrease until it all but plateaus. I wonder if you could bring up this concept or find a citation (perhaps in vocal repertoires) in which sampling effort and number of new observed signals was demonstrated to further substantiate your point.

This is a great analogy, and we have now included it in our paper: “But as with hours of video footage, increasing the number of individuals may not necessarily lead to more unique facial configurations, especially if a repertoire maximum has been reached. One analogy that can be used to illustrate the relationship between hours of video footage, number of sampled individuals, and number of identified facial configurations is the sampling efforts associated with obtaining measures of species richness. The greater the sampling effort, the more individuals are collected, and the higher the probability of identifying rare species until a maximum is reached (Batista et al., 2021). This could also apply to facial signals: the greater the sampling effort, the more facial signals are collected, and the greater the probability of identifying new facial configurations until a maximum is reached” (lines 118-127).

Line 86: I think more nuance could be useful here. Increasing more animals may influence the signals observed, but what may be even more influential would be including more individuals of different life stages (i.e. juveniles vs adults, males vs. females, etc.)

This is a very good point! We have addressed it by adding the following two sentences: “Previous studies on the facial signaling repertoires of chimpanzees (Florkiewicz et al., 2023), hylobatids (Scheider et al., 2014; Scheider et al., 2016; Florkiewicz et al., 2018; Florkiewicz et al., 2023), and domesticated cats (Scott & Florkiewicz et al., 2023) have primarily focused on adults. When increasing the number of individuals to sample from, it may be beneficial to sample from a diverse range of ages and sexes to create a more representative facial signaling repertoire of the species” (lines 113-118).

After reading your results, however, I would suggest to the authors to perhaps reframe how they introduce the ideas in this paragraph. As it is written, I was expecting for the authors to collect new datasets with more individuals and more contact hours, whereas they actually run a simulation in which they generate a theoretical maximum of possible combinations. I therefore think that some discussion of theoretical combinatorial possibilities and how sampling effort could influence detecting these would be useful.

We have added a new preceding paragraph to address this idea: “However, it is unclear whether these numbers represent all the unique facial signals that each mammal can generate. When envisioning facial muscle movements as numbers and communicative facial configurations as permutations, the potential facial signaling repertoire of a given species becomes quite extensive. Even when repetition is not allowed, the number of possible facial signals generated is still quite large. For instance, previous research has indicated that the average number of distinct facial muscle movements in a given facial signal is around 3 for chimpanzees, hylobatids, and domesticated cats (Florkiewicz et al., 2023; Scott & Florkiewicz et al., 2023). If we calculate the number of permutations for a subset of 15 facial muscle movements while discouraging repetition and selecting 3 movements each time, the result is 2,730 possible facial signals. This estimate of the number of morphologically distinct facial signals already exceeds what has been previously documented for all three species (Florkiewicz et al., 2023; Scott & Florkiewicz et al., 2023). But this estimate may be generous, as it does not consider the relationship between individual facial muscle movements, such as muscle movements that must occur together or are mutually exclusive. Even when implementing these relationships, the range of facial signals may still be extensive. It is possible that challenges related to behavioral sampling have led to smaller documented repertoires.” (lines 84-99).

Lines 196-199: I appreciate that you are using previously published datasets. However, the difference in sampling hours for these two species, especially in light of your discussion in the introduction about the potential consequences of sampling effort, is potentially concerning. It may be good to explicitly acknowledge this limitation.

We acknowledge this limitation by adding the following sentence at the end of this paragraph: “Because there are difference in the number of recording hours for both species, we cannot directly compare the observed configuration lists, as the identification rates would not be the same” (lines 193-195).

Line 233-234: it is not entirely clear to me from the description why you would eliminate movements observed in the chimpanzee dataset but not in the cat dataset if your intention was to document all possible potential facial movements. You are not comparing chimpanzees to cats as far as I understand, but observed to possible within chimpanzees and cats. Therefore, I am not certain this titration of your chimpanzee dataset is necessary, especially since it appears as though the cat dataset retained ear movements which are certainly not present in the chimpanzee datasets.

We have added the following sentence to emphasize our rationale for omitting these extra movements from our chimpanzee dataset: “Because it is highly likely that cats also make these kinds of movements, and that these movements could influence their facial signaling repertoire size, we chose to exclude them from the chimpanzee dataset to ensure comparability of our observed configuration lists” (lines 223-226).

Data analysis section: I would appreciate a bit more justification as to why this analysis is being run and what these p values would represent. At present, I am not confident I am interpreting the rationale behind these analyses as the authors intend, and would like clarification. However, as I see it, it appears to be a means of identifying whether there are any particular facial movements that are more or less represented in combinations in the observed vs. possible datasets?

After receiving feedback from another anonymous reviewer (who expressed concerns with pseudoreplication), we decided to omit the t-tests and p-values and focus on qualitative comparisons instead. We have modified both tables to provide additional information about differences in the proportions of observed facial muscle movements and clarified why we made these comparisons (and what they mean) in our discussion section (lines 427-458).

Line 392: I think this is the core of your paper, but also one that, at present, I am not sure you are quite at answering. I think the first step to this would be to identify theoretical maxima, as your Python script seems capable. However, what I would like to see is a subsampling procedure from your observed chimpanzee dataset in which you perhaps took an increment of 3 hours (to match that of the cats), 10, 50, 100, 150 or however many or few steps you would like and identify how many combinations you observed at each sample size. Your resultant plot would look like a species richness curve in which you could very clearly see whether you are approaching an asymptote or only observing more and more combinations as you increase sampling effort. If you were to add this to your combination generation procedure, then I think you could demonstrate not only that there may be more theoretical combinations, but also perhaps demonstrate a minimum sampling duration or at the least show the effect of increasing sampling effort.

Due to concerns about pseudoreplication and the lack of data on the number of distinct facial configurations identified per hour (as this information was not logged), we have decided to restructure the paper to be more qualitative in nature. We now acknowledge that the lack of comparable video recording hours is a limitation of our study (lines 193-195). Additionally, we have clarified our stance on why our Python models can be a valuable tool for researchers in reallocating research efforts in the discussion section (lines 515-530).

Line 427: I am not sure I understand this sentence in light of the previous one, which describes non-facial expression behaviors. Do the authors mean morphologically distinct multimodal postures/behaviors?

We have clarified this sentence (and the preceding one) to refocus our discussion on morphologically distinct facial signals: “However, our estimates regarding the number of possible facial configurations for each species may change when including additional facial action descriptors (such eye movements, head positions, and head movements). When these additional facial muscle movements and descriptors are considered, the number of morphologically distinct facial signals in chimpanzees, for example, increases from 66 to 357 (Florkiewicz et al., 2023)” (lines 411-416).

There are two points not considered in the discussion which I would like to see mention:

1) the effect of context on the proportion of signals observed. For instance, it is mentioned that nose wrinkle, a subtle behavior, was not observed as frequently as could be theoretically possible. Could it be that certain facial movements are less combinatorially flexible than others due to the context in which they are produced. For instance, if nose wrinkle is used to disambiguate and signal non-affiliative aggression, I would not expect to see it in many combinations, even if theoretically it could be; and
   a. We have added the following paragraph to address this point: “However, it is possible that differences in the proportion of identified facial muscle movements in our “observed” and “unobserved” configuration lists signify differences in their social function rather than underreporting due to coding error. Previous studies with chimpanzees, for example, found that certain facial muscle movements are associated with particular types of facial signals that often have strong associations with a given context (Parr et al., 2007). For example, AU10 (Upper Lip Raiser) is typically associated with the production of scream faces, and seldom appears in other types of communicative displays (like play faces and pouts; Parr et al., 2007). In contrast, other facial muscle movements (such as AU12) appear across multiple facial signal types (such as bared-teeth displays, play faces, and scream faces; Parr et al., 2007). In our current research, we noticed that AU10 appeared more frequently in unobserved facial configurations than in observed ones, but the opposite pattern was observed for AU12. This pattern could have been observed because AU12 has multiple communicative functions, while AU10 is limited in its communicative functioning. Additional research should focus on discerning the communicative function of discrete facial muscle movements, in addition to the function of facial configurations, to explore this possibility further” (lines 500-514).
2) the distinction between theoretically possible vs. not actually communicative combinations. This comes down to how animals actually use communication, and whether there are conventional/meaning constraints on the possible combinations that actually are used.
   a. We have added the following paragraph to address this point: “It is important to note that in order to determine whether a facial configuration is communicative, the behavioral responses of recipients must be evaluated. If signals help to elicit behavioral responses in recipients that have positive fitness consequences for both signalers and recipients (Laidre & Johnstone, 2013), then there should be observable behavioral responses from recipients. This may also involve a clear attempt to elicit a behavioral response from the signaler's perspective, with evidence of intentionality and/or goal-directed behavior (Scheider et al., 2014; Byrne et al., 2017). It is only by coding the behavioral responses of both signalers and recipients that we can determine which configurations generated by our Python models are communicative or non-communicative. Increasing sample sizes and hours of video footage may reveal additional combinations that are used as facial signals, but it is also possible that our “observed” datasets represent the full communicative potential of each species.” (lines 376-386).

**Minor comments**:
Line 86: “it’s” to “it is”. It is best practice to avoid contractions in formal writing.

Thank you! We have removed instances of it’s and replaced them with “it is” throughout the manuscript.

Line 106: to clarify, you are not really hoping to compile a comprehensive list of all possible facial expressions for all mammals, but only for chimpanzees and cats. I think rewording this sentence is necessary to more accurately reflect the scope of the current study.

We have reworked the first sentence and added additional information for clarity: “In our current study, we aim to assess how well the existing literature represents the possible range of facial signaling among mammals. To achieve this aim, we will compile a comprehensive list of all possible facial muscle movement combinations (referred as facial configurations from this point onward) for mammals with established FACS and previously documented facial signaling repertoires” (lines 144-148).

Line 128: I suggest rewording to “we will hereafter refer to them as ‘facial cue/signal’.”

Because another reviewer suggested replacing "facial cue/signal" with "facial configurations" and shortening the "current study" section for clarity, we have omitted this sentence.

Line 233: missing period after the closing parenthetical.

Thank you for catching this! We have fixed it.

Fig. 1: I would suggest removing the brown outline around the cat’s pupils, as it looks a bit too much like a human-like iris. I also suggest writing in the text itself what each AU is rather than just indicating the number.

After carefully considering this suggestion and evaluating the overall representation of the facial muscle movements in the illustration, we have decided to exclude Figure 1. We believe that the images appear too anthropomorphic, even after removing the brown outline around the cat's pupils.

Table 1: I would suggest naming what these AUs are rather than just numbering them to aid in comprehension for people who either are not fluent in FACs or study a system in which some of these AUs are absent and therefore unfamiliar. I also recommend bolding the significant comparisons and adding asterisk to aid in rapidly identifying these values.

We have added a new column that includes the full descriptions of each AU/AD. We have replaced the last column with information on differences in proportions of observed muscle movements between observed and unobserved configuration lists.
